# Rediscovery of *Mazus lanceifolius* reveals a new genus and a new species in Mazaceae

**DOI:** 10.3897/phytokeys.171.61926

**Published:** 2021-01-06

**Authors:** Chun-Lei Xiang, Hong-Li Pan, Dao-Zhang Min, Dai-Gui Zhang, Fei Zhao, Bing Liu, Bo Li

**Affiliations:** 1 CAS Key Laboratory for Plant Diversity and Biogeography of East Asia, Kunming Institute of Botany, Chinese Academy of Sciences, Kunming 650201, Yunnan, China Kunming Institute of Botany, Chinese Academy of Sciences Kunming China; 2 Sichuan Academy of Forestry, Chengdu 610081, Sichuan, China Sichuan Academy of Forestry Chengdu China; 3 Research Centre of Ecological Sciences, College of Agronomy, Jiangxi Agricultural University, Nanchang 330045, China Jiangxi Agricultural University Nanchang China; 4 Key Laboratory of Plant Resources Conservation and Utilization, Jishou University, Jishou 416000, China Jishou University Jishou China; 5 State Key Laboratory of Systematic and Evolutionary Botany, Institute of Botany, Chinese Academy of Sciences, Beijing 100093, China Institute of Botany, Chinese Academy of Sciences Beijing China; 6 Sino-African Joint Research Center, Chinese Academy of Sciences, Wuhan 430074, China Sino-African Joint Research Center, Chinese Academy of Sciences Wuhan China

**Keywords:** *
Dodartia
*, Lamiales, *
Lancea
*, new genus, *
Puchiumazus
*

## Abstract

*Mazus lanceifolius* (Mazaceae) is a perennial herb with opposite leaves and endemic to central China that has not been collected for 130 years. Rediscovery of this enigmatic species in the wild allows for determination of its phylogenetic position within Mazaceae. Phylogenetic reconstruction of Mazaceae based on DNA sequences from four plastid markers (*matK*, *rbcL*, *rps16* and *trnL*-*trnF*) and nuclear ribosome ITS consistently showed that *Mazus* was not monophyletic. *Mazus lanceifolius* is in the most basal clade within Mazaceae, as sister to the remaining species of three recognized genera *Dodartia*, *Lancea* and *Mazus*. These results support the separation of *M. lanceifolius* from *Mazus* as a new genus, which was established here as *Puchiumazus* Bo Li, D.G. Zhang & C.L. Xiang. Meanwhile, a collection from Shennongjia Forestry District of Hubei Province, China, misidentified as “*M. lanceifolius*” in previous molecular study, is here revealed to represent an undescribed species of *Mazus*, i.e., *M. fruticosus* Bo Li, D.G. Zhang & C.L. Xiang, **sp. nov.** Morphologically, *Puchiumazus* is clearly distinct from the other three genera by having quadrangular to somewhat ribbed stems, and obviously opposite leaves. In addition, we provide a taxonomic key to the four genera of Mazaceae.

## Introduction

Mazaceae (Reveal 2011) is a small herbaceous family in Lamiales currently containing three genera: *Dodartia* L., *Lancea* Hook.f. & Thomson and *Mazus* Lour. (APG IV 2016; Olmstead 2016; Christenhusz et al. 2017). The monotypic genus *Dodartia* based on *D. orientalis* L., occurs mainly in southern Russia and western to central Asia (Fischer 2004) and is characterized by having scale-like leaves and much-branched stems. The genus *Lancea* is found only in the Qinghai-Tibetan Plateau (QTP) where it includes two species, *L. tibetica* Hook.f. & Thomson and *L. hirsuta* Bonati (Chi et al. 2018, 2019), of which the former species is widely used in traditional Tibetan medicine. Morphologically, *Lancea* is characterized by leaves in a rosette and a lower corolla lip with a distinct palate. *Mazus* is the largest genus in Mazaceae, including approximately 30 species of annual or perennial herbs (Hong et al. 1998; Deng et al. 2016) distributed in Asia, Australia and New Zealand (Li 1954; Barker 1991; Fischer 2004). China is considered to be the center of distribution and differentiation of the genus (Yang 1979; Hsieh 2000), with ca. 26 species and three varieties currently recorded (Hong et al. 1998; Deng et al. 2016). Species delimitation in *Mazus* has been problematic because of relatively high levels of morphological variation (Li 1954; Hong et al. 1998). In general, *Mazus* can be distinguished from the other two genera by a combination of morphological characters: a strongly two-lipped corolla (3/2-bilabiatae), a palate with two longitudinal plaits and a capsule enveloped in a persistent calyx (Fischer 2004; Deng et al. 2019).

*Dodartia*, *Lancea* and *Mazus* were once placed in the traditionally circumscribed Scrophulariaceae (e.g. Von Wettstein 1891) but variably affiliated with tribe Gratioleae (Von Wettstein 1891; Thieret 1954, 1967) or Mimuleae (Dumortier 1829; Burtt 1965; Argue 1984; Fischer 2004). However, Scrophulariaceae were found to be polyphyletic and some genera were subsequently transferred to existing families like Orobanchaceae, Plantaginaceae, Phrymaceae and Stilbaceae, and some genera were separated as small monophyletic families, including Calceolariaceae, Linderniaceae, Mazaceae, Paulowniaceae, Schlegeliaceae, and Wightiaceae (Olmstead and Reeves 1995; Oxelman et al. 1999, 2005; Olmstead et al. 2001; Beardsley and Olmstead 2002; Albach et al. 2005; Rahmanzadeh et al. 2005; Tank et al. 2006; Schäferhoff et al. 2010; Liu et al. 2020), then leaving a much reduced Scrophulariaceae s.s. To date, a number of genera have not yet been sequenced and are still unplaced.

When redefining Phrymaceae based on molecular phylogenetics, Beardsley and Olmstead (2002) had first shown that *Mazus* and *Lancea* formed a well-supported group that was weakly supported as sister to the rest of Phrymaceae. Consequently, they tentatively included the two genera in the redefined Phrymaceae and assigned them to a provisional subfamily “Mazoideae” (Beardsley and Olmstead 2002). However, subsequent studies did not recover the sister relationship between “Mazoideae” and the rest of Phrymaceae, and *Mazus* and *Lancea* were found to be sister to the Orobanchaceae+Paulowniaceae+Phrymaceae clade (Oxelman et al. 2005; Albach et al. 2009; Schäferhoff et al. 2010). Thus, a new family MazaceaeReveal (2011) was established to accommodate this. When *Dodartia* was first included in a molecular analysis, Xia et al. (2012) found that this genus was closely related to *Lancea* and they together formed the sister clade of *Mazus*. Currently, MazaceaeReveal (2011) with the inclusion of all these three genera has been widely accepted (Refulio-Rodriguez and Olmstead 2014; APG IV 2016; Olmstead 2016; Christenhusz et al. 2017). It was found to be a member of the clade comprising Lamiaceae, Mazaceae, Wightiaceae, Phrymaceae, Paulowniaceae and Orobanchaceae (Liu et al. 2020).

**Figure 1.**
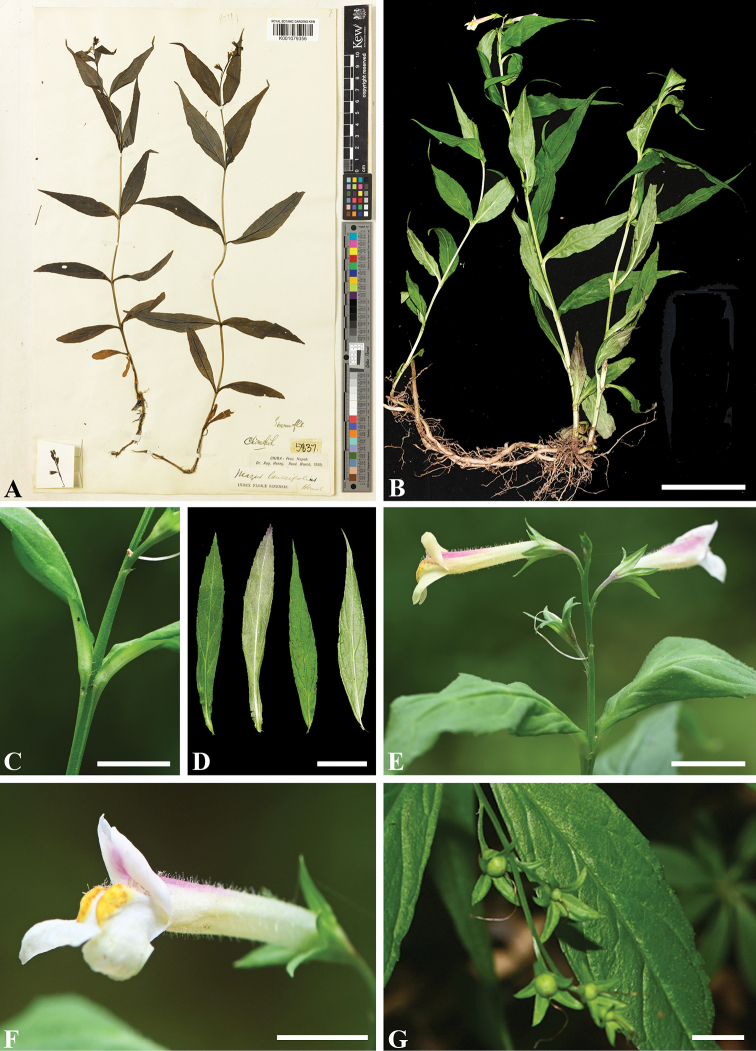
*Puchiumazus lanceifolius* (≡ *Mazus lanceifolius*) **A** lectotype deposited at K (*A. Henry 5837*, barcode K001079356) **B** habit **C** stem, showing the obtuse ribs **D** leaves **E** inflorescence **F** flower in lateral review **G** young fruits. Scale bars: 5 cm (**B**); 0.5 cm (**C, F, G**); 2 cm (**D**); 1 cm (**E**).

Within the genus *Mazus*, *M. lanceifolius* Hemsl. is a distinctive species through its quadrangular stems and narrowly lanceolate, mostly cauline, opposite leaves (Fig. 1). By contrast, the other species of *Mazus* have terete stems and leaves often in basal rosettes (Yang 1979; Hong et al. 1998). Therefore, *M. lanceifolius* was assigned to a monotypic section: sect. LanceifoliaeBonati (1908), which was followed by Yang (1979). Since its description by Forbes and Hemsley (1890), *M. lanceifolius* has never been recorded by any specimens until two populations of the rare species were rediscovered in Sichuan Province of China in 2020. The rediscovery of *M. lanceifolius* after more than one century offers us a precious opportunity to test its phylogenetic position based on morphological and molecular data.

**Figure 2.**
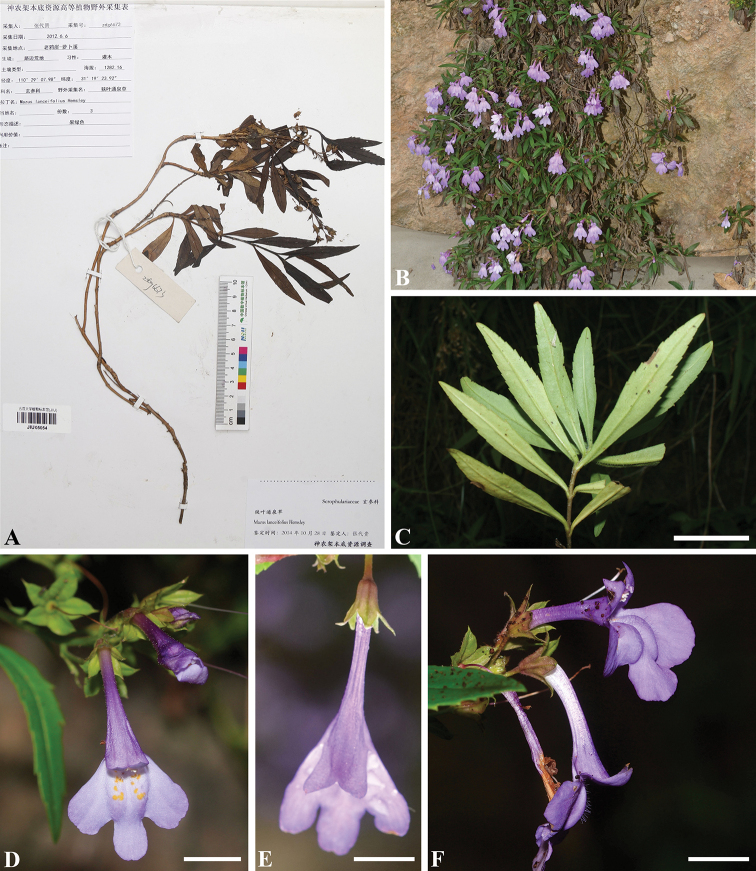
*Mazus fruticosus***A** voucher of “*Mazus lanceifolius*” sampled in Deng et al. (2019), deposited at JIU (the herbarium of Jishou University, Hu’nan, China) **B** habit and habitat **C** leaves **D** flower in frontal view, showing morphology of its lower lips **E** flower in frontal view, showing morphology of its upper lips **F** flowers in lateral view. Scale bars: 2 cm (**C**); 0.5 cm (**D, E, F**).

Since the establishment of the family Mazaceae (Reveal 2011), only one molecular phylogenetic study exclusively focused on its phylogeny (Deng et al. 2019), including one species from each *Lancea* and *Dodartia*, and 23 out of 30 species of *Mazus*. In that study, Deng et al. (2019) notably included two samples named as “*Mazus lanceifolius*”, and stated that “*M. lanceifolius*” can be easily distinguished from other *Mazus* species by having lanceolate leaves and a robust stem. After consulting the vouchers of “*Mazus lanceifolius*” (*D.G. Zhang zdg6673*, Fig. 2) sampled by Deng et al. (2019) as well as the type specimens (*Henry 7250*, K001079356!; *Henry 5837*, K001079356!) and the original description of *M. lanceifolius*, we found that the plants of “*Mazus lanceifolius*” used by Deng et al. (2019) have opposite to subopposite leaves, which may have led the authors to identify the plant as *M. lanceifolius* because this species is the only known *Mazus* species with opposite leaves. However, except for these opposite leaves, their “*Mazus lanceifolius*” is remarkably different from the type specimen of *M. lanceifolius* in many aspects. For example, the plants sampled by Deng et al. (2019) are robust shrubs having numerous and much branched stems, leathery leaves that are acutely serrate on the apical half and multiflowered inflorescences (Fig. 2; see also fig. 2C in Deng et al. 2019), while the type material of *M. lanceifolius* is a slender herb having several unbranched stems, submembranaceous and almost entire leaves and remarkably sparse inflorescences with no more than six flowers (Fig. 1). We therefore have to conclude that the specimen sampled as “*M. lanceifolius*” by Deng et al. (2019) was misidentified, with the identity of that sample needing to be confirmed.

In the present study, we carried out an updated phylogeny of Mazaceae, in order to (1) investigate the phylogenetic placement of the distinct and enigmatic species *M. lanceifolius* based on its rediscovered populations; (2) confirm the identity of the misidentified *M. lanceifolius* by Deng et al. (2019); and (3) further contribute to a comprehensive phylogenetic framework for Mazaceae.

## Material and methods

### Field work, taxon sampling and data collection

Two populations of *Mazus lanceifolius* were rediscovered in June 2020 in Sichuan Province, China. One is located in the Qingchengshan Mountain near Dujiangyan City, and another was found in Qianfoshan Mountain near Mianyang City. Morphological observations were conducted based on wild individuals as well as the type specimens. Fresh leaves were collected in the field and dried with silica-gel for DNA extraction (Chase and Hills 1991). Voucher specimens are deposited in the herbarium of Shanghai Chenshan Botanical Garden (**CSH**).

In the present study, most DNA sequences are based on previous phylogenetic analyses (Deng et al. 2019), but some problematic sequences were excluded for analyses. For example, the *trnL*-*trnF* sequences of *Mazus japonicus* (Thunb.) Kuntze 3 (KX807207) in the study of (Deng et al. 2019) were actually under the name of *M. pumilus* (Burm. f.) Steenis in GenBank. Similarly, *trnL*-*trnF* sequences of two different species (i.e. *Mazus* sp., MK266435 and Mazus japonicus var. delavayi (Bonati) P.C. Tsoong, KX783521) are completely identical. Such kinds of sequences were excluded for analyses. In addition, two individuals of *Dodartia orientalis* and three individuals of *Lancea tibetica* were included for analyses. Thus, all genera (*Mazus*, *Lancea* and *Dodartia*) of the newly established family Mazaceae (Reveal 2011) were represented. Voucher information and GenBank accession numbers for taxa used in this study are provided in Appendix 1.

Based on previous studies (Schäferhoff et al. 2010; Refulio-Rodriguez and Olmstead 2014; Luna et al. 2019; Xia et al. 2019; Liu et al. 2020), 14 taxa representing 12 genera in five families (*Pedicularis* L., *Rehmannia* Libosch. ex Fisch. & C.A. Mey. and *Striga* Lour. [Orobanchaceae], *Paulownia* Siebold & Zucc. [Paulowniaceae], *Erythranthe* Spach, *Mimulus* L. and *Phryma* L. [Phrymaceae], *Wightia* Wall. [Wightiaceae], *Callicarpa* L., *Lamium* L., *Premna* L. and *Vitex* L. [Lamiaceae]) were selected as outgroups for the cpDNA dataset. While, because of the high divergence of nrITS sequences, only eight species from the above-mentioned families were selected as outgroups.

### DNA extraction, amplification and sequencing

Total genomic DNA was obtained from silica-dried leaves using the CTAB procedure of Doyle and Doyle (1987). After extraction, the DNA was re-suspended in double-distilled water and kept at -40 °C for polymerase chain reaction (PCR) amplifications.

The DNA amplifications were performed in a thermocycler (Eppendorf Scientific, Inc., Westbury, NY, USA). Based on Deng et al. (2019), four cpDNA regions (*matK*, *rbcL*, *rps16* and *trnL*-*trnF*) and nrITS were selected for phylogenetic reconstruction. Primers, protocols for PCR, sequencing followed those in Deng et al. (2019) and references therein.

### Phylogenetic analysis

Sequences were initially assembled and edited with Geneious v.7.1.7 (Kearse et al. 2012) and aligned using MUSCLE (Edgar 2004) as implemented in Geneious v.7.1.7 (Kearse et al. 2012). The final alignments were manually adjusted in PhyDe v.0.9971 (Müller et al. 2010). The four chloroplast DNA regions were combined directly because the plastid genome is mostly uniparentally inherited (Soltis and Soltis 1998) and supposedly safe to be combined in phylogenetic analyses (Olmstead and Sweere 1994). Nuclear (ITS) and the combined plastid data set were analyzed separately using maximum likelihood (ML) and Bayesian inference (BI) methods.

ML analyses were performed using RAxML-HPC2 v.8.2.9 (Stamatakis 2014) as implemented on the CIPRES Science Gateway (http://www.phylo.org/) (Miller et al. 2010) under the GTRGAMMA model. The partitioned model (-q) was used for the concatenated plastid data, bootstrap iterations (-# | -N) set to 1000, and other parameters followed default settings.

BI analyses using Markov chain Monte Carlo (MCMC) methods (Yang and Rannala 1997) were performed with MrBayes v3.2.2 (Ronquist et al. 2012) and implemented on the CIPRES Science Gateway (http://www.phylo.org/) (Miller et al. 2010). The optimal substitution models were selected using Model Finder (Kalyaanamoorthy et al. 2017) plugin in PhyloSuite (Zhang et al. 2018). Model parameters were estimated directly during the runs. For each Bayesian analysis, four MCMC chains were run simultaneously for 20 million generations. Each run began with one random tree and sampled one tree every 1000 generations. At the end of the run, chain convergence and estimated sample size (ESS) parameters were assessed with Tracer v.1.6.0 (Rambaut et al. 2014). A 50% majority-rule consensus tree was calculated for each dataset after discarding the first 25% of the trees as burn-in. In the resulting summary tree, posterior probability values (PP) ≥ 0.95 were considered to be strongly supported (Suzuki et al. 2002).

## Results

### Sequence and alignment characterization

Ten sequences were newly generated for this study (Appendix 1). The resulting combined and aligned cpDNA dataset contained 4514 positions (including gaps), of which 1287 positions belong to *matK*, 1266 to *rbcL*, 963 to the *rps16* partition and 998 to the *trnL*-*trnF* spacer. Of these 1259 (27.89%) nucleotides were variable in the dataset (Table 1). The aligned nrITS dataset includes 641 nucleotides, of which 300 (46.80%) were variable (Table 1).

**Table 1.** Properties and best-fitting models of data partitions used in this study.

Data matrix	Aligned positions	Variable characters	GC content (%)	AIC selected model
*matK*	1287	431	33.4%	GTR+F+G4
*rbcL*	1266	172	43.8%	GTR+F+I+G4
*rps16*	963	333	33.4%	GTR+F+G4
*trnL*-*trnF*	998	323	35.4%	GTR+F+G4
Combined cpDNA matrix	4514	1259	37.2%	GTR+F+I+G4
nrITS	641	300	60.1%	GTR+F+I+G4

### Phylogenetic analysis of Mazaceae

**Figure 3.**
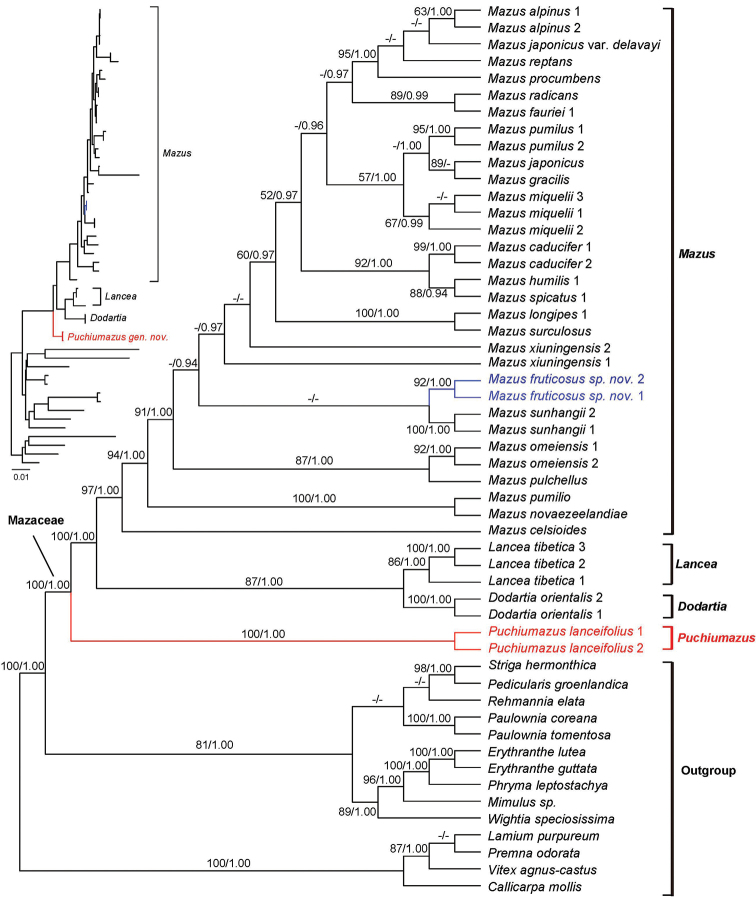
Maximum Likelihood phylogram of Mazaceae as inferred from analysis of combined dataset of *matK*, *rbcL*, *rps16* and *trnL*-*trnF*. Support values ≥ 50% BS or 0.90 PP are displayed near the branches following the order ML-BS/BI-PP.

**Figure 4.**
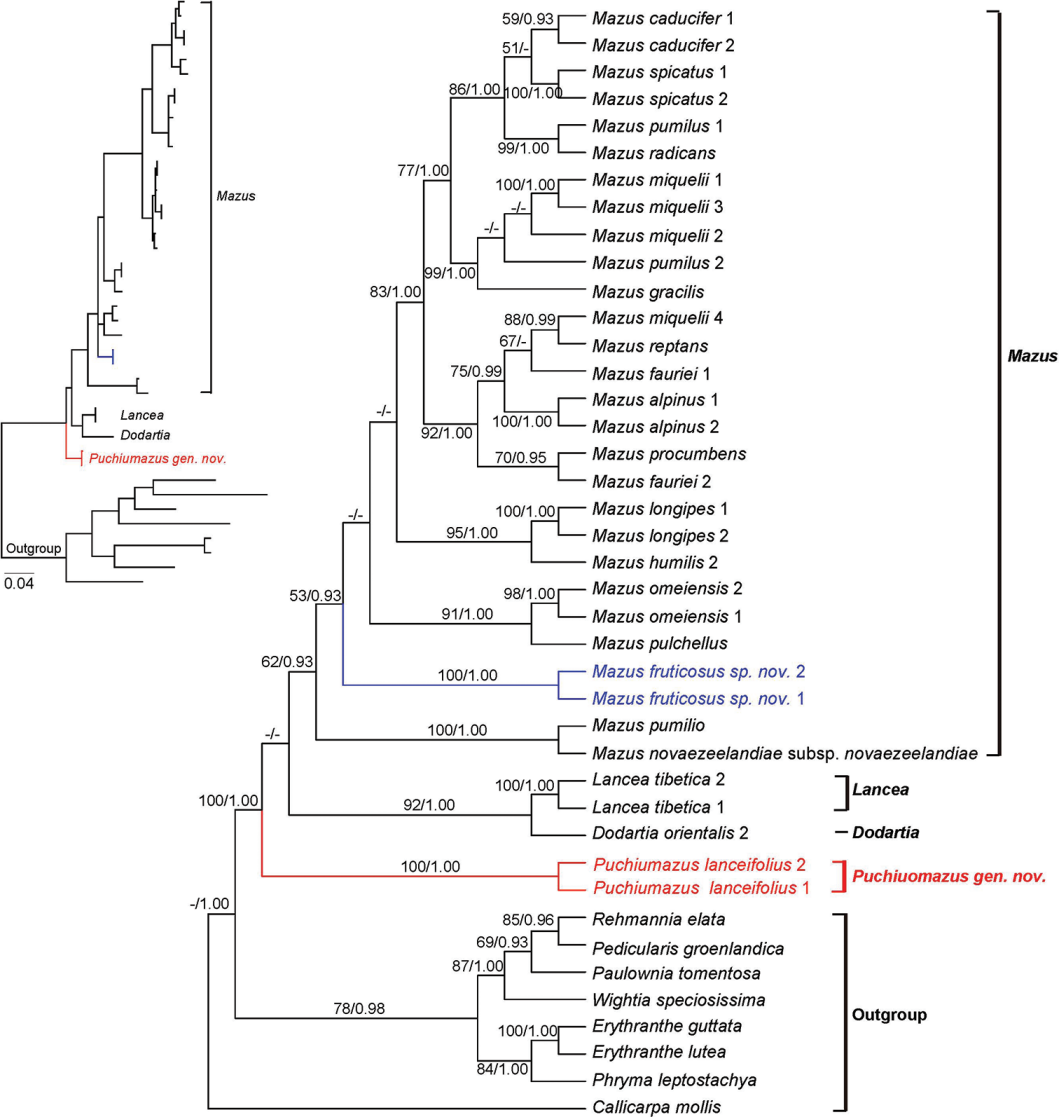
Maximum Likelihood phylogram of Mazaceae as inferred from analysis of nrITS. Support values ≥ 50% BS or 0.90 PP are displayed near the branches following the order ML-BS/BI-PP.

In all analyses, the monophyly of Mazaceae was strongly supported (Figs 3, 4; ML BS: 100%, BIPP: 1.00; all values reported in this order below). Because the taxon sampling is different in the datasets of cpDNA and nrITS, we did not combine them for analyses.

Three subclades can be identified in the cpDNA (Fig. 3) as well as nrITS trees (Fig. 4). The two individuals of *M. lanceifolius* consistently form a clade sister to the rest of Mazaceae. Within the rest of the family, *Dodartia*-*Lancea* clade is sister to *Mazus* (Figs 3, 4). In both ML and BI analyses, a sister relationship between *Lancea* and *Dodartia* is well supported (87%, 1.00 in cpDNA tree; 92%, 1.00 in nrITS tree). Monophyly of *Mazus* is also strongly supported (97%, 1.00) based on cpDNA dataset while moderately supported in nrITS analyses (62%, 0.93). Relationships within the genus *Mazus* are not fully resolved (Figs 3, 4). The “*M. lanceifolius*” misidentified in Deng et al. (2019) was found to be grouped with *M. sunhangii* based on cpDNA analyses with low support values (Fig. 3), while emerging as an isolated lineage in nrITS analyses when ITS sequence of *M. sunhangii* was not available (Fig. 4).

### Taxonomic treatment

#### Puchiumazus

Taxon classificationPlantaeLamialesMazaceae

Bo Li, D.G. Zhang & C.L. Xiang gen. nov.

DCD544F7-882B-5134-A45E-67C4A97C8B3D

urn:lsid:ipni.org:names:77213610-1

Fig. 1

##### Type.

*Puchiumazus lanceifolius* (Hemsl.) Bo Li, D.G. Zhang & C.L. Xiang ≡ *Mazus lanceifolius* Hemsl., in: *J. Linn. Soc.*, *Bot.* 26 (174): 181. 1890.

##### Diagnosis.

**Figure 5.**
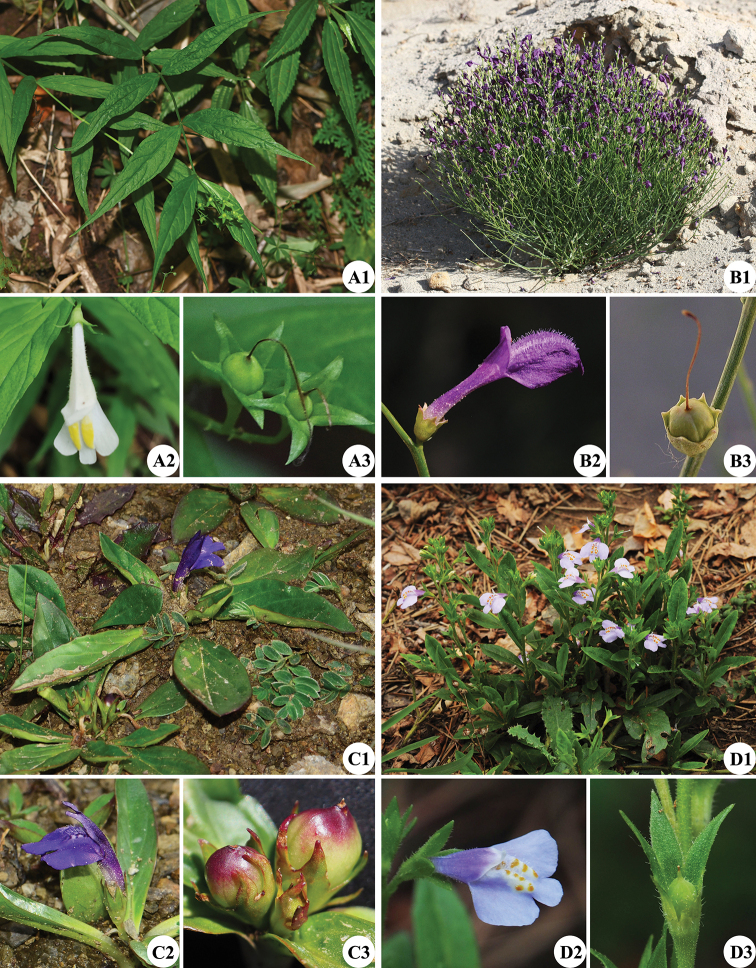
Morphological comparisons of the four genera of Mazaceae**A***Puchiumazus lanceifolius***B***Dodartia orientalis***C***Lancea tibetica***D***Mazus stachydifolius***A1, B1, C1, D1** habits **A2, B2, C2, D2** flowers **A3, B3, C3, D3** fruits.

The new genus is characterized by having quadrangular to somewhat ribbed stems and opposite, narrowly lanceolate leaves (Figs 1, 5A1–A3). *Puchiumazus* is sister to a clade composed of *Dodartia*, *Lancea* and *Mazus*. Morphologically, it is most similar to *Mazus*, but it differs in having quadrangular stems, lanceolate leaves (vs. terete stems and usually obovate-oblong leaves).

##### Description.

Perennial herbs. Rhizomes fleshy, white, horizontal. Root thin, fibrous. Stems erect, unbranched, glabrous, up to 30 cm tall, old stems quadrangular, glabrous, young stems inconspicuously quadrangular to obtusely ribbed, minutely puberulent. Leaves opposite, petiole inconspicuous to nearly absent; leaf blade narrowly lanceolate, 5.5–8.5 × 0.8–1.1 cm, submembranaceous to papery, adaxially green, pubescent, abaxially pale green, (sub)glabrous, base cuneate, margin basally entire and apically sparsely serrate, apex acute to long acuminate; lateral veins 3–5 pairs, abaxially raised and adaxially slightly depressed. Racemes terminal, 3–6 cm, flowers remarkably sparse, less than 6; pedicels 4–7 mm, sparsely puberulent; bracts tiny, narrowly lanceolate to linear. Calyx funnelform, 4–6 mm, sparsely pubescent outside, subglabrous inside, 5-lobed; lobes narrowly triangular to lanceolate, as long as tube in length, midrib conspicuous, apex acute. Corolla creamy yellow, 1.8–2.2 cm long, densely puberulent outside; tube straight, cylindric, long exserted from calyx, gradually dilated; limb 2-lipped, reddish in throat, posterior lip bilobed, lobes orbicular, anterior lip trilobed, lobes subequal, rounded. Stamens 4, didynamous, inserted on corolla tube, included, anterior pair longer; anthers bithecal, locules divergent, apically connivent; filaments filiform, glabrous. Styles included, glabrous, persistent; stigma 2-lamellate. Capsule ovoid, ca. 2 × 3 mm, glabrous.

##### Etymology.

The generic name is derived from “*Puchiu*” (in honor of Prof. Pu Chiu Tsoong (1906–1981), who was a prominent Chinese taxonomist specializing in the taxonomy of Scrophulariaceae in the traditional sense) and “*mazus*”, indicating that the new genus was separated from *Mazus* and is morphologically similar to it.

##### Common name

**(assigned here).** Bu Qiu Cao Shu (补求草属; Chinese name).

##### Distribution.

According to our data, this genus is endemic to Central China. It is known only from Hubei (Jianshi), Sichuan (Dayi and Dujiangyan) and Chongqing (Wushan) and can be found under evergreen broad-leaf forest at elevations of 600–1250 m.

#### Puchiumazus lanceifolius

Taxon classificationPlantaeLamialesMazaceae

(Hemsl.) Bo Li, D.G. Zhang & C.L. Xiang comb. nov.

AC9D277B-FE44-51A8-978A-6BE20EFE0D72

urn:lsid:ipni.org:names:77213611-1

Fig. 1

Mazus lanceifolius Hemsl., in: *J. Linn. Soc.*, Bot. 26(174): 181. 1890. Lectotype (**designated here**): China. Hubei province (Hupeh): Jianshi (Chienchih), March 1889, *A. Henry 5837* (K barcode K001079356 [photo!]). Basionym.

##### Phenology.

Flowering and fruiting from March to July.

##### Common name

**(assigned here).** Bu Qiu Cao (补求草; Chinese name).

##### Additional specimens examined.

China. Sichuan Province (Szechuen): South Wushan, March 1889, *A. Henry 7250* (K barcode K001079357 [photo!]); Duajiangyan City, Qingchengshan Mountain, under evergreen broad-leaf forest, 1200 m elev., 3 June 2020, *X.X. Zhou et al. LB1067*; Mianyang City, Dayi County, Qianfoshan Mountain, 850 m elev., 8 June 2020, *X.X. Zhou et al. LB1067-2*.

##### Note.

In the protologue of *Mazus lanceifolius*, two collections from Sichuan (*A. Henry 7250*) and Hubei (*A. Henry 5837*), China, respectively, were simultaneously listed without exact type designation because that was not the practice in the 19^th^ century. After checking all floras and literature dealing with *Mazus* in China, we are certain that *M. lanceifolius* has not been lectotypified before. Thus, we here propose the specimen *A. Henry 5837* (Kew barcode: K001079356) as lectotype of *M. lanceifolius* (Fig. 1A) in accordance with article 9.3 of the *International Code of Nomenclature for Algae, Fungi, and Plants* (*Shenzhen Code*) (Turland et al. 2018).

#### Mazus fruticosus

Taxon classificationPlantaeLamialesMazaceae

Bo Li, D.G. Zhang & C.L. Xiang, sp. nov.

A8045804-DA4F-544B-84CC-588B1DC71F0D

urn:lsid:ipni.org:names:77213612-1

Fig. 2

##### Type.

China. Hubei Province: Shennongjia Forestry District, Laoyaya to Luoboxi, on rocky cliffs, 110°29'07.98"N, 31°19'23.92"E, 1282 m elev., 6 June 2012, *D.G. Zhang zdg6673* (Holotype: JIU!).

##### Diagnosis.

*Mazus fruticosus* differs from all other conspecific taxa by being a shrub with numerous and much branched stems and having opposite to subopposite leathery leaves that are acutely serrate on apical half.

##### Description.

Shrubs, 25–55 cm tall. Stems woody, numerous branched, old stems greyish brown, terete, leafless, glabrous, young stems and branchlets brown, densely puberulent. Leaves nearly fascicled on the top of branchlet, opposite to subopposite, subsessile; lamina lanceolate, leathery, 3.5–5.5 × 0.7–1.1 cm, adaxially green, subglabrous to sparsely puberulent, abaxially light green, subglabrous, puberulent on veins, apex acute to acuminate, base cuneate, margin acutely serrate on apical half; midrib conspicuous abaxially, lateral veins inconspicuous; petioles nearly absent, densely puberulent. Racemes terminal, ascending to 7.5 cm long, lax, multiflowered; pedicels slender, 1–1.5 cm long, puberulent; bracts narrowly lanceolate, 3–4 mm long, puberulent. Calyces broadly campanulate, ca. 5 mm long, slightly enlarged in fruit, 5-veined, pubescent outside, pubescent to subglabrous inside; lobes 5, broadly triangular, as long as tube, apex acute, midrib conspicuous, lateral veins inconspicuous. Corolla purple, dotted yellow on palate, 1.6–1.9 cm long, puberulent to subglabrous outside, tube cylindric, 1.1–1.3 cm long, exserted from calyx; limb 2-lipped, upper lip bilobed, erect, lobes triangular ovate; lower lip trilobed, middle lobe narrowly ovate, ca. 3 mm long, smaller than lateral lobes, lateral lobes spreading away from middle lobe, broadly ovate to rectangular; palate comprising 2 longitudinal elevations extending from point of filament fusion to base of lower lobes, with sparse erect hairs. Stamens 4, didynamous, glabrous, inserted at the same level in distal part of tube, included; anterior pair longer, curved, appressed to corolla tube, posterior pair spreading; anthers bithecal, positioned adjacent to corolla tube on upper lip; filaments filiform, glabrous. Styles 1.4–1.7 cm long, included, exserted beyond anthers, stigma 2-lamellate. Capsule globose, ca. 4 mm in diam, apex rounded, included by persistent calyx.

##### Etymology.

The epithet of the new species refers to its shrubby habit.

##### Common name

**(assigned here).** Guan Zhuang Tong Quan Cao (灌状通泉草; Chinese name).

##### Distribution and habitat.

*Mazus fruticosus* is currently known only from Shenlongjia Forest District in Hubei Province, central China. It frequently occurs on rocky cliffs or near evergreen mixed forests at an elevation of 1100–1250 m.

##### Additional specimens examined.

China. Hubei Province: Shennongjia Forestry District, 29 March 2012, *D.G. Zhang y1071* (JIU!); 11 May 2012, *D.G. Zhang zdg00023* (JIU!); 17 August 2012, *D.G. Zhang 00006* (JIU!); 21 May 2013, *D.G. Zhang 130521012* (JIU!); 23 April 2015, *D.G. Zhang 0423007* (JIU!).

### Key to the four genera of Mazaceae

**Table:** 

1	Stems quadrangular or somewhat ribbed; leaves opposite	*** Puchiumazus ***
–	Stems not quadrangular; leaves rosette, alternate or rarely opposite to subopposite	**2**
2	Stems much branched; leaves reduced, scale-like; lower corolla lip without palate	*** Dodartia ***
–	Stems inconspicuous or unbranched, rarely much branched in *Mazus*; Leaves not reduced; lower lip with distinct palate	**3**
3	Fruit usually completely enclosed in calyx when mature	*** Mazus ***
–	Fruit half enclosed by calyx when mature	*** Lancea ***


## Discussion

We here reconstruct the phylogeny of Mazaceae based on a combined cpDNA dataset of four markers (*matK*, *rbcL*, *rps16* and *trnL*-*trnF*), and nrDNA ITS dataset, which have been used previously to infer relationships within Mazaceae (Deng et al. 2019; Yamamoto 2020) and among Lamiales (Refulio-Rodriguez and Olmstead 2014; Liu et al. 2020). The monophyly of Mazaceae is recovered as reported in previous work (Deng et al. 2019) relying on the same molecular markers. The major difference is that the third clade identified in the present study was not sampled by Deng et al. (2019).

Based on our analyses (Figs 3, 4), Mazaceae is composed of four genera (Fig. 5), including the new genus *Puchiumazus* described here. Three major clades can be identified for a re-circumscribed Mazaceae, and the cladogram is accompanied by some general morphological characters and geographical distribution patterns. The first clade is composed of two individuals of the new monotypic genus *Puchiumazus* (Figs 1, 5A1–A3), which is currently only known from three provinces in central China. Morphologically, the new genus can be distinguished clearly from other genera by having quadrangular to somewhat ribbed stems and opposite, narrowly lanceolate leaves.

The second clade consists of *Dodartia* (Fig. 5B1–B3) and *Lancea* (Fig. 5C1–C3). Both genera have broader distribution area than *Puchiumazus*, with *Lancea* always found at high elevations in QTP and *Dodartia* distributed in southern Russia and western to central Asia; it is cultivated as medical herb which has increased its distribution. Morphologically, both genera have small scale-like leaves (with a basal rosette of larger leaves in *Lancea*). Another important character is that ca. half of the capsule is enclosed by fruiting calyx and that calyx-teeth are much shorter than the fruit (Fig. 5B3, C3). In *Puchiumazus*, the style is persistent and ca. 2/3 of the fruit is enclosed in the fruiting calyx with calyx-teeth being much longer than the fruit. Calyx of *Mazus* is usually at least 1–2 times longer than capsule (e.g., Fig. 5D3).

Species of *Mazus* comprise the third clade, which is well supported in the cpDNA tree (94%, 1.00; Fig. 3), but moderately supported in the nrITS phylogeny (62%, 0.93; Fig. 4). *Mazus* is the largest genus of Mazaceae and it is widely distributed in East Asia and Australia. It can be distinguished from the other three genera by the more or less secund inflorescences and a corolla with a palate on the lower lip. Using the same DNA markers, Deng et al. (2019) produced a fully resolved phylogeny of *Mazus* in which five clades of the genus were highly supported (see Fig. 4 of their study). The interesting finding is that we cannot recover a similar topology, although the data of most species come from their dataset. Part of the reason for this may be that some sequences generated for their study were wrongly submitted to GenBank (see samples in Material and methods). Another possible reason is that they did not consider the topology incongruence between cpDNA and nrITS sequences, but concatenated the data for their analyses.

Phylogenetic analyses in our study did not support the sectional classification (i.e. *Lanceifoliae*, *Mazus* and *Trichogymus*) of *Mazus* proposed by Hong et al. (1998). At that time, *Mazus lanceifolius* was placed within *Mazus*, which we here recognize as a new genus. In addition, monophyly of the remaining two sections was also not supported, which was also the case in the study of Deng et al. (2019). Accordingly, they proposed a new infrageneric classification of *Mazus*, with two subgenera, *Mazus* and Notomazus T. Deng, N. Lin & H. Sun. Subgenus Mazus comprises most of the species and is native to Asia, while subgenus Notomazus comprises all species native to Australia and New Zealand. However, the monophyly of the two subgenera were not supported in our study. In both cpDNA and nrITS trees, *Mazus radicans* (Hook.f.) Cheeseman from subgenus Notomazus is deeply nested in subgenus Mazus, indicating it is necessary to redefine subgenus Notomazus. Given the discordance between the trees presented here and the one presented in Deng et al. (2019), on the basis of the same sequence data, we think some additional checking of the data, perhaps even resampling of *M. radicans*, is needed before any revision is made to the subgeneric classification of *Mazus*. In addition, a future study including more individuals of each species and more DNA markers (especially single and/or low copy nuclear genes) is necessary to clarify internal relationships within *Mazus*.

Previously, all species of *Mazus* are described as herbs (Yang 1979; Hong et al. 1998; Fischer 2004), but five species (*M. caducifer* Hance, *M. celsioides* Hand.-Mazz., *M. spicatus* Vaniot, “*M. lanceifolius*” [described as *M. fruticosus* in the present study], and *sp.*) were recorded as having “no herbaceous stem” in Deng et al.’s (2019) study. Actually, *M. caducifer*, *M. spicatus*, *M. celsioides* have rigid stems that look woody, but are not actually forming wood, thus these should be recognized as having a herbaceous habit. The new species described in the present study is probably the only species with a shrubby habit in the genus *Mazus*. This interesting find will help us to better understand the character evolution of *Mazus*. If *Mazus sp.* in Deng et al.’s (2019) also has a shrubby habit, we can speculate this character originated independently at least twice within the genus.

The abovementioned findings mean that more intensive field collections are necessary even in the post-Flora time. Yang (1979) have noticed the morphological difference between *Puchiumazus lanceifolius* (≡ *Mazus lanceifolius*) and other *Mazus* species. He pointed out that the quadrangular stem is only found in this species, and the nearly entire lanceolate leaves are also rare in *Mazus*, thus he suggested that this species probably is generically distinct. At the same time, he also emphasized that, because no fully developed flowers could be investigated based on specimens, he placed this species within *Mazus*. In this study, the rediscovery of this species offers an opportunity to investigate morphological characters of *P. lanceifolius* and provide a chance to extract DNA for molecular phylogenetic analyses, which led to the establishment of the new genus in the present study.

In recent years, many plants of Lamiales were rediscovered from biodiversity hotspots of China, including *Aeschynanthus monetaria* Dunn (Gesneriaceae; Hu et al. 2020), *Ombrocharis dulcis* Hand.-Mazz. (Lamiaceae; Chen et al. 2016), *Wenchengia alternifolia* C.Y. Wu & S. Chow (Lamiaceae; Li et al. 2012) and *Pedicularis humilis* Bonati (Orobanchaceae; Li et al. 2016). Most of these species had only been collected once before. The new genus described in the present study was also only known from the type collections (*A. Henry 5837*, *7250*) before it was rediscovered. The type specimens of this were, until recently, the only known collections, and as a result, studies on the species since the original 1890 publication have been wanting. The re-investigation of this species is not only providing a chance to amend its description, but also a chance for a recognition of a new genus and redefinition of the family. The study highlights the important roles of field collections for systematic and biodiversity studies, which are often neglected in this age of biodiversity informatics (Wen et al. 2015).

## Supplementary Material

XML Treatment for Puchiumazus

XML Treatment for Puchiumazus lanceifolius

XML Treatment for Mazus fruticosus

## Appendix 1

**Table A1.** Source publications and GenBank accession numbers of DNA sequences used in this study. If papers were not published, then indicated using superscript, references were listed below the table. GenBank accession numbers of the newly sequenced are marked in bold face. An n-dash (–) refers to a missing sequence.

Taxon	References	GenBank No.				
		*matK*	*rbcL*	*rps16*	*trnL–trnF*	ITS
**Ingroups**						
*Dodartiao rientalis* 1	Schäferhoff et al. (2010)	FN773539	–	FN794091	FN794057	–
*Dodartiao rientalis* 2	Deng et al. (2019)	MK392230	JQ342984	JQ342982	JQ342981	JQ342980
*Lancea tibetica* 1	Deng et al. (2019)	MK266276	KX783467	KX807200	KX807205	MK192678
*Lancea tibetica* 2	Xia et al. (2009); Zuniga et al. (2017)^a^	MF786907 ^a^	MF786661 ^a^	FJ172699	FJ172685	FJ172736
*Lancea tibetica* 3	Chi et al. (2018)	MF593117	MF593117	MF593117	MF593117	–
*Mazus reptans*	Refulio-Rodriguez and Olmstead (2014); Beardsley and Olmstead (2002)	HQ384502	HQ384872	HQ385147	AF479004	AF478940
*Mazus alpinus* 1	Deng et al. (2019)	MK266256	KX783481	KX783501	KX783520	MK192641
*Mazus alpinus* 2	Deng et al. (2019)	–	KX783480	KX783500	KX783519	MK192642
*Mazus caducifer* 1	Deng et al. (2019)	MK266277	KX783477	KX783497	KX783516	MK192664
*Mazus caducifer* 2	Deng et al. (2019)	–	KX783487	KX783506	KX783526	MK192659
*Mazus celsioides*	Deng et al. (2019)	–	KX783486	MK266366	KX783525	–
*Mazus fauriei* 1	Deng et al. (2019)	MK266255	–	KX783499	MK266420	MK192640
*Mazus fauriei* 2	Deng et al. (2019)	–	–	–	–	LC034207
*Mazus gracilis*	Xia et al. (2009)	–	FJ172729	FJ172701	FJ172687	FJ172738
*Mazus humilis* 1	Deng et al. (2019)	–	–	MK266367	MK266421	–
*Mazus humilis* 2	Deng et al. (2019)	–		–	–	MK192667
Mazus japonicus var. delavayi	Deng et al. (2019)	MK266257	KX783482	KX783502	KX783521	–
*Mazus japonicas*	Xia et al. (2009); Deng et al. (2019)	MK266259	FJ172728	FJ172700	FJ172686	–
*Mazus fruticosus* 1	Deng et al. (2019)	MK266261	KX783470	KX783490	KX783509	MK192660
**Appendix 1 Continued**						
*Mazus fruticosus* 2	Deng et al. (2019)	MK266254	KX783471	KX783491	KX783510	MK192649
*Mazus longipes* 1	Deng et al. (2019)	MK266267	KX783474	KX783494	KX783513	MK192652
*Mazus longipes* 2	Deng et al. (2019)	–	–	–	–	MK192654
*Mazus miquelii* 1	Deng et al. (2019)	–	KX783475	KX783495	KX783514	MK192637
*Mazus miquelii* 2	Deng et al. (2019)	MK266271	KX783476	KX783496	KX783515	MK192655
*Mazus miquelii* 3	Deng et al. (2019)	MK266272	KX783483	KX783503	KX783522	MK192656
*Mazus miquelii* 4	Umemoto et al. (2015)	–	–	–	–	LC027734
*Mazus novaezeelandiae*	Deng et al. (2019)	MK266278	KX783469	KX783489	KX783508	MK192676
*Mazus omeiensis* 1	Deng et al. (2019)	MK266252	KX807209	KX807203	KX807208	MK192636
*Mazus omeiensis* 2	Xia et al. (2009); Deng et al. (2019)	–	FJ172731	FJ172702	FJ172688	MK192663
*Mazus pulchellus*	Deng et al. (2019)	–	KX783472	KX783492	KX783511	MK192638
*Mazus pumilus* 1	Deng et al. (2019); Jiang et al. (2018)^b^; Xu et al. (2018)^c^	MH265198 ^b^	MK266346	KX807201	KX807206	MH711724 ^c^
*Mazus pumilus* 2	Xia et al. (2009); Schaefer et al. (2011); Deng et al. (2016)	HM850959	HM850162	KX807202	KX807207	FJ172737
*Mazus pumilio*	Deng et al. (2019)	MK266277	KX783468	KX783488	KX783507	MK192671
*Mazus radicans*	Deng et al. (2019); Smissen et al. (2015)^d^	–	KT626738 ^d^	MK266381	–	MK192635
*Mazus spicatus* 1	Xia et al. (2009)	MK266251	FJ172730	FJ172703	FJ172689	FJ172740
*Mazus spicatus* 2	Deng et al. (2019)	–	–	–	–	MK192681
*Mazus surculosus*	Deng et al. (2019)	–	KX783473	KX783493	KX783512	–
*Mazus sunhangii* 1	Deng et al. (2016)	–	KX783485	KX783505	KX783524	–
*Mazus sunhangii* 2	Deng et al. (2016)	–	KX783484	KX783504	KX783523	–
*Mazus xiuningensis* 1	Deng et al. (2019)	–	MK266348	MK266383	–	–
*Mazus xiuningensis* 2	Deng et al. (2019)	–	MK266349	MK266384	MK266430	–
*Mazus procumbens*	Deng et al. (2019)	MK266261	KX783478	KX783498	KX783517	MK192647
*Puchiumazus lanceifolius* 1	This study	**MW373735**	**MW373737**	**MW373739**	**MW373741**	**MW364623**
*Puchiumazus lanceifolius* 2	This study	**MW373736**	**MW373738**	**MW373740**	**MW373742**	**MW364624**
**outgroups**						
*Paulownia tomentosa*	Xu et al. (2018)^c^; Deng et al. (2019)	MK392226	KX783466	KX807199	KX807204	MH711291 ^c^
*Paulownia coreana*	Yi and Kim (2016)	NC_031435	NC_031435	NC_031435	NC_031435	–
*Lamium purpureum*	Wink and Kaufmann (1996); Oxelman et al. (2005); Refulio-Rodriguez and Olmstead (2014)	HQ384493	Z37403	HQ385141	AJ608588	–
*Callicarpa mollis*	Tsukaya et al. (2003); Refulio-Rodriguez and Olmstead (2014)	HQ384498	HQ384868	HQ385145	HQ412928	AB099648
*Vitex agnus*–*castus*	Refulio-Rodriguez and Olmstead (2014); Wagstaff and Olmstead (1997)	HQ384496	U78716	HQ385143	HQ412926	–
*Premna odorata*	Refulio-Rodriguez and Olmstead (2014)	HQ384494	HQ384866	HQ385142	HQ412925	–
*Wightia speciosissima*	Xia et al. (2019); Zhou et al. (2014)^e^	MK381318	MK381318	MK381318	MK381318	KJ563189 ^e^
*Mimulus* sp.	Zhao et al. (2021)	MT473772	MT473772	MT473772	MT473772	–
*Phryma leptostachya*	Wagstaff and Olmstead (1997); Bremer et al. (2002); Xu et al. (2018)^c^	AJ429341	U28881	AJ609150	AJ430928	MH711667 ^c^
*Erythranthe lutea*	Vallejo-Marín et al. (2016); Arroyo et al. (2019)	NC_030212	NC_030212	NC_030212	NC_030212	MH781192
*Erythranthe guttata*	Refulio-Rodriguez and Olmstead (2014); Kuzmina et al. (2017)	KJ161979	KJ161981	KJ161978	KJ161975	MG219646
*Striga hermonthica*	Wicke et al. (2016)	KU212372	KU212372	KU212372	KU212372	–
**Appendix 1 Continued**						
*Rehmannia elata*	Oxelman et al. (2005); Albach et al. (2006); Refulio-Rodriguez and Olmstead (2014)	HQ384505	HQ384874	DQ856490	AJ608572	DQ069315
*Pedicularis groenlandica*	Refulio-Rodriguez and Olmstead (2014); Tkach et al. (2014)	HQ384503	HQ384873	HQ385148	HQ412930	HG424130

**References in Appendix**

^a^ Zuniga JD, Mulcahy DG, Coddington JA (2017) DNA barcodes from Tibetan plant genera. [Unpublished.]

Albach DC, Li HQ, Zhao N, Jensen SR (2006) Molecular systematics and phytochemistry of *Rehmannia* (Scrophulariaceae). Biochemical Systematics and Ecology 35(5): 293–300. https://doi.org/10.1016/j.bse.2006.11.003

Arroyo MTK, Pérez F, Jara-Arancio P, Pacheco D, Vidal P, Flores MF (2018) Ovule bet-hedging at high elevation in the South American Andes: Evidence from a phylogenetically controlled multispecies study. Journal of Ecology 107(2): 668–683. https://doi.org/10.1111/1365-2745.13069

^b^ Jiang CC, Zhang CC, Wei SS, Huang HH, Huang ZZ (2018) Identification of Weeds Based on DNA Barcoding. [Unpublished.]

Beardsley PM, Olmstead RG (2002) Redefining Phrymaceae: The placement of *Mimulus*, tribe Mimuleae, and *Phryma*. American Journal of Botany 89(7): 1093–1102. https://doi.org/10.3732/ajb.89.7.1093

Bremer B, Bremer K, Heidari N, Erixon P, Olmstead RG, Anderberg AA, Källersjö M, Barkhordarian E (2002) Phylogenetics of asterids based on 3 coding and 3 non-coding chloroplast DNA markers and the utility of non-coding DNA at higher taxonomic levels. Molecular Phylogenetics and Evolution 24(2): 274–301. https://doi.org/10.1016/S1055-7903(02)00240-3

^c^ Xu Y, Zeng CX, Zhang YQ, Ren Y, Ge XJ (2018) DNA barcoding the Flora of Qinling Mt. in China. [Unpublished.]

Chi X, Wang J, Gao Q, Zhang F, Chen SL (2018) The complete chloroplast genomes of two *Lancea* species with comparative analysis. Molecules (Basel, Switzerland) 23(3): e602. https://doi.org/10.3390/molecules23030602

^d^ Smissen RD, Millar RT, Heenan P (2015) Phylogenetic Tree of New Zealand vascular genera. [Unpublished.]

Deng T, Zhang XS, Kim C, Zhang JW, Zhang DG, Volis S (2016) *Mazus sunhangii* (Mazaceae), a new species discovered in Central China appears to be highly endangered. PLoS ONE 11(10): e0163581. https://doi.org/10.1371/journal.pone.0163581

Deng T, Lin N, Huang X, Wang H, Kim C, Zhang D, Zhu W, Yusupov Z, Tojibaev ShK, Sun H (2019) Phylogenetics of Mazaceae (Lamiales), with special reference to intrageneric relationships within *Mazus*. Taxon 68(5): 1037–1047. https://doi.org/10.1002/tax.12150

^e^ Zhou QM, Jensen SR, Liu GL, Wang S, Li HQ (2014) Familial placement of *Wightia* (Lamiales). [Unpublished.]

Kuzmina ML, Braukmann TWA, Fazekas AJ, Graham SW, Dewaard SL, Rodrigues A, Bennett BA, Dickinson TA, Saarela JM, Catling PM, Newmaster SG, Percy DM, Fenneman E, Lauron-Moreau A, Ford B, Gillespie L, Subramanyam R, Whitton J, Jennings L, Metsger D, Warne CP, Brown A, Sears E, Dewaard JR, Zakharov EV, Hebert PDN (2017) Using herbarium-derived DNAs to assemble a large-Scale DNA barcode library for the vascular plants of Canada. Applications in Plant Sciences 5(12): e1700079. https://doi.org/10.3732/apps.1700079

Oxelman B, Kornhall P, Olmstead RG, Bremer B (2005) Further disintegration of Scrophulariaceae. Taxon 54(2): 411–425. https://doi.org/10.2307/25065369

Refulio-Rodriguez NF, Olmstead RG (2014) Phylogeny of Lamiidae. American Journal of Botany 101(2): 287–299. https://doi.org/10.3732/ajb.1300394

Schaefer H, Hardy OJ, Silva L, Barraclough TG, Savolainen V (2011) Testing Darwin’s naturalization hypothesis in the Azores. Ecology Letters 14(4): 389–396. https://doi.org/10.1111/j.1461-0248.2011.01600.x

Schäferhoff B, Fleischmann A, Fischer E, Albach DC, Borsch T, Heubl G, Müller KF (2010) Towards resolving Lamiales relationships: Insights from rapidly evolving chloroplast sequences. BMC Evolutionary Biology 10(1): e352. https://doi.org/10.1186/1471-2148-10-352

Tkach N, Ree RH, Kuss P, Röser M, Hoffmann MH (2014) High mountain origin, phylogenetics, evolution, and niche conservatism of arctic lineages in the hemiparasitic genus *Pedicularis* (Orobanchaceae). Molecular Phylogenetics and Evolution 76: 75–92. https://doi.org/10.1016/j.ympev.2014.03.004

Tsukaya H, Eukuda T (2003) Hybridization and introgression between *Callicarpa japonica* and *C. mollis* (Verbenaceae) in central Japan, as inferred from nuclear and chloroplast DNA sequences. Molecular Ecology 12(11): 3003–3011. https://doi.org/10.1046/j.1365-294X.2003.01961.x

Umemoto H, Yokota M, Kokubugata G (2015) Reconsideration for Occurrence of *Mazus goodenifolius* (Phrymaceae) in Miyazaki Prefecture, Japan using Molecular and Morphological Data. Bulletin of the National Museum of Nature and Science, Series B 41: 61–67.

Vallejo-Marín M, Cooley AM, Lee MY, Folmer M, McKain MR, Puzey JR (2016) Strongly asymmetric hybridization barriers shape the origin of a new polyploid species and its hybrid ancestor. American Journal of Botany 103(7): 1272–1288. https://doi.org/10.3732/ajb.1500471

Wagstaff SJ, Olmstead RG (1997) Phylogeny of Labiatae and Verbenaceae inferred from *rbcL* sequences. Systematic Botany 22(1): 165–179. https://doi.org/10.2307/2419684

Wicke S, Müller KF, DePamphilis CW, Quandt D, Bellot S, Schneeweiss GM (2016) Mechanistic model of evolutionary rate variation en route to a nonphotosynthetic lifestyle in plants. Proceedings of the National Academy of Sciences of the United States of America 113(32): 9045–9050. https://doi.org/10.1073/pnas.1607576113

Wink M, Kaufmann M (1996) Phylogenetic relationships between some members of the subfamily Lamioideae (Family Labiatae) inferred from nucleotide sequences of the *rbcL* gene. Botanica Acta 109(2): 139–148. https://doi.org/10.1111/j.1438-8677.1996.tb00554.x

Xia Z, Wang YZ, Smith JF (2009) Familial placement and relations of *Rehmannia* and *Triaenophora* (Scrophulariaceae s.l.) inferred from five gene regions. American Journal of Botany 96(2): 519–530. https://doi.org/10.3732/ajb.0800195

Xia Z, Wen J, Gao Z (2019) Does the enigmatic *Wightia* belong to Paulowniaceae (Lamiales)? Frontiers of Plant Science 10: e528. https://doi.org/10.3389/fpls.2019.00528

Yi DK, Kim KJ (2016) Two complete chloroplast genome sequences of genus *Paulownia* (Paulowniaceae): *Paulownia coreana* and *P. tomentosa*. Mitochondrial DNA. Part B, Resources 1(1): 627–629. https://doi.org/10.1080/23802359.2016.1214546

Zhao F, Chen YP, Salmaki Y, Drew BT, Wilson TC, Scheen AC, Celeop F (accepted for publication) Bräuchler c, Bendiksby M, Wang Q, Ming DZ, Peng H, Olmstead RG, Li B, Xiang CL (2021) An updated tribal classification of Lamiaceae based on plastome phylogenomics. BMC Biology. https://doi.org/10.1186/s12915-020-00931-z

## Supplementary materials

Supplementary material 1 Figures S1, S2 https://doi.org/10.3897/phytokeys.171.61926.suppl1 Data type Phylogenetic tree Explanation note **Figure S1.** Bayesian Inference (BI) phylogram of Mazaceae based on the combined cpDNA dataset (*matK*, *rbcL*, *rps16*, and *trnL-F*). Bayesian posterior probabilities are shown near the branches. **Figure S2.** Bayesian Inference (BI) phylogram of Mazaceae based on the nrITS dataset. Bayesian posterior probabilities are shown near the branches. https://binary.pensoft.net/file/494562This dataset is made available under the Open Database License (http://opendatacommons.org/licenses/odbl/1.0/). The Open Database License (ODbL) is a license agreement intended to allow users to freely share, modify, and use this Dataset while maintaining this same freedom for others, provided that the original source and author(s) are credited.Chun-Lei Xiang, Hong-Li Pan, Dao-Zhang Min, Dai-Gui Zhang, Fei Zhao, Bing Liu, Bo Li
