# Supplementary figures and images for: Rediscovery of Mazus lanceifolius reveals a new genus and a new species in Mazaceae
Source: PhytoKeys. 2021 Jan 6;171:1–24. doi: 10.3897/phytokeys.171.61926 (PMC7806577; doi:10.3897/phytokeys.171.61926)

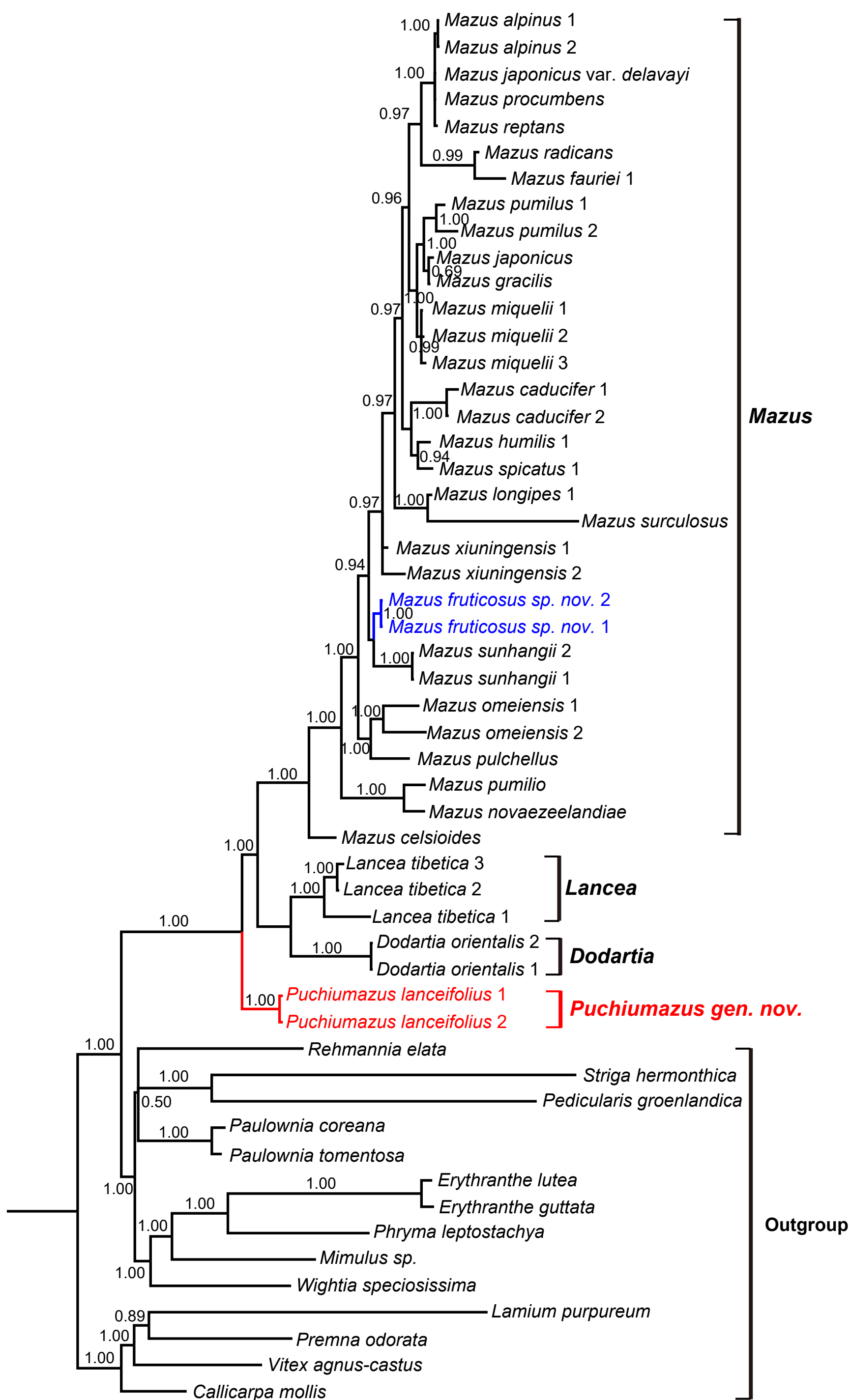

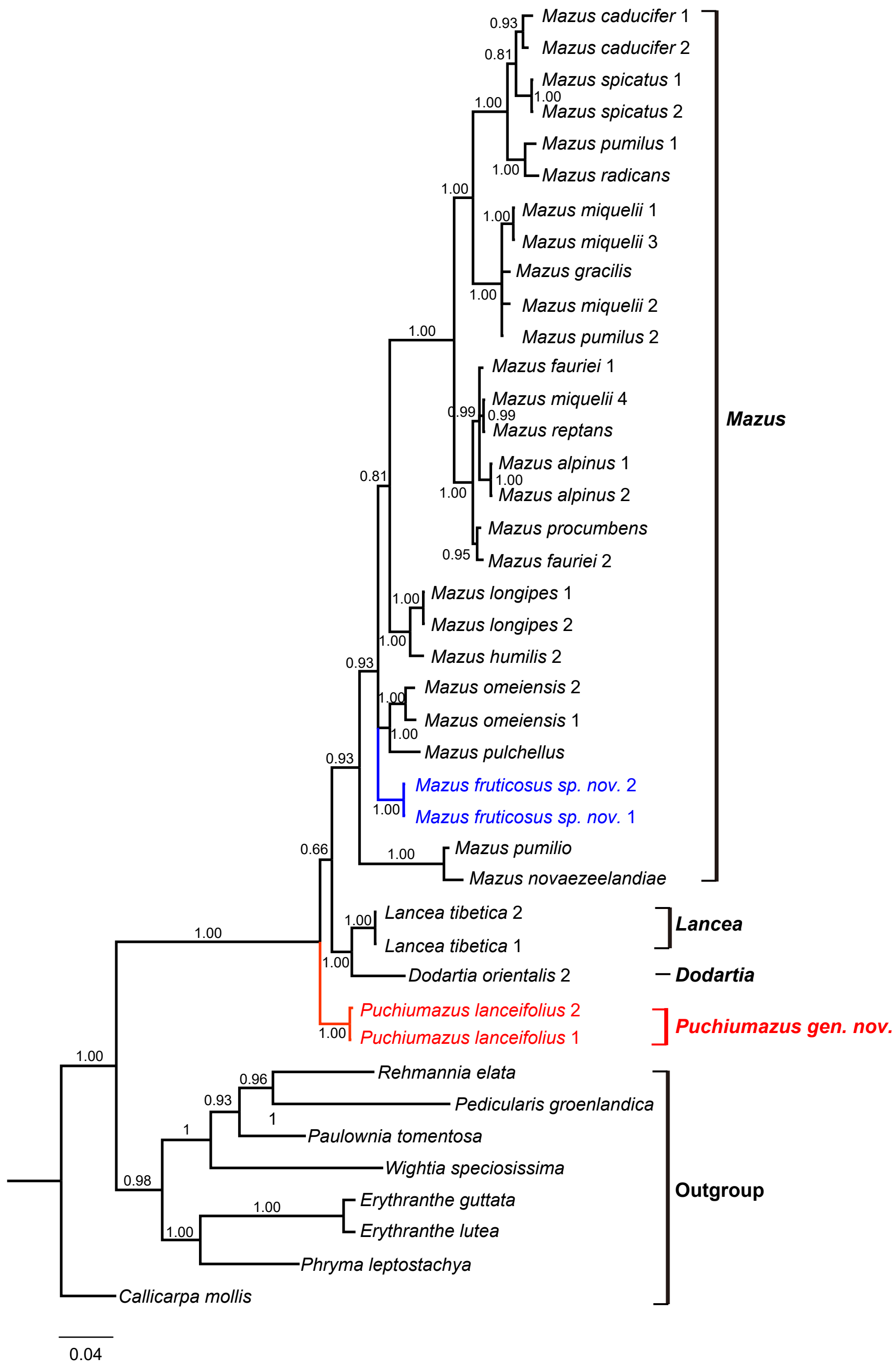

Supplement: Supplementary material 1 — Figures S1, S2 [file phytokeys-171-001-s001.pdf]
